# Cranial Musculoskeletal Description of Black-Throated Finch (Aves: Passeriformes: Estrildidae) with DiceCT

**DOI:** 10.1093/iob/obab007

**Published:** 2021-04-30

**Authors:** K H T To, H D O’Brien, M R Stocker, P M Gignac

**Affiliations:** Department of Geosciences, Virginia Tech, Derring Hall, 926 W Campus Dr, Blacksburg, VA 24060, USA; Department of Anatomy and Cell Biology, Oklahoma State University Center for Health Sciences, 1111 W 17th Street, Tulsa, OK 74107, USA; Department of Geosciences, Virginia Tech, Derring Hall, 926 W Campus Dr, Blacksburg, VA 24060, USA; Department of Anatomy and Cell Biology, Oklahoma State University Center for Health Sciences, 1111 W 17th Street, Tulsa, OK 74107, USA

## Abstract

Synopsis Dietary requirements and acquisition strategies change throughout ontogeny across various clades of tetrapods, including birds. For example, birds hatch with combinations of various behavioral, physiological, and morphological factors that place them on an altricial–precocial spectrum. Passeriformes (=songbirds) in particular, a family constituting approximately more than half of known bird species, displays the most drastic difference between hatchling and adults in each of these aspects of their feeding biology. How the shift in dietary resource acquisition is managed during ontogeny alongside its relationship to the morphology of the feeding apparatus has been largely understudied within birds. Such efforts have been hampered partly due to the small size of many birds and the diminutive jaw musculature they employ. In this study, we used standard and diffusible iodine-based contrast-enhanced computed tomography in conjunction with digital dissection to quantify and describe the cranial musculature of the Black-throated Finch (*Poephila cincta*) at fledgling and adult stages. Our results reveal that in both the fledgling and the adult, cranial musculature shows clear and complex partitioning in the *Musculus adductor mandibulae externus* that is consistent with other families within Passeriformes. We quantified jaw-muscle sizes and found that the adult showed a decrease in muscle mass in comparison to the fledgling individual. We propose that this could be the result of low sample size or a physiological effect of parental care in Passeriformes. Our study shows that high-resolution visualization techniques are informative at revealing morphological discrepancies for studies that involve small specimens such as Passeriformes especially with careful specimen selection criteria.

## Abstract (trans abstract)

Nhiều nhánh động vật có tứ chi, bao gồm cả chim, có nhu cầu dinh dưỡng và chiến thuật kiếm ăn liên tục thay đổi xuyên suốt quá trình sinh trưởng của chúng. Ví dụ, chim non nở dưới sự tác động của các yếu tố hành vi, sinh lý, và hình thái để xác định thời điểm chúng có thể tự kiếm ăn. Bộ Sẻ *Passeriformes*, một bộ bao gồm hơn phân nửa số loài chim hiện nay, thể hiện sự chênh lệch lớn nhất về các yếu tố kể trên trong tập tính kiếm ăn của cá thể mới sinh và cá thể trưởng thành. Làm sao sự thay đổi trong cách kiếm ăn được kiểm soát trong quá trình sinh trưởng, cũng như quan hệ của nó với cấu tạo hàm, là những chủ đề còn ít được quan tâm ở chim. Các nỗ lực nghiên cứu trước đây thường bị giới hạn một phần bởi kích thước nhỏ của vô số loài chim, đi kèm với hệ cơ hàm còn nhỏ hơn của chúng. Trong nghiên cứu này, chúng tôi sử dụng phương pháp chụp cắt lớp vi tính tiêu chuẩn (CT) và với chụp cắt lớp tăng cường độ tương phản dựa trên i-ốt khuếch tán (diffusible iodine-based contrast-enhanced computed tomography, diceCT), bên cạnh với việc giải phẫu kỹ thuật số, để định lượng và mô tả hệ cơ đầu của loài Di cổ đen (Black-throated Finch, *Poephila cincta*) ở giai đoạn rời tổ và khi trưởng thành. Kết quả phân tích cho thấy, ở cả chim non mới rời tổ và chim trưởng thành, hệ cơ đầu có sự phân chia phức tạc và rõ rệt tại bó cơ cắn ngoài (*Musculus adductor mandibulae externus*), tương tự các họ chim khác trong bộ Sẻ. Khi định lượng kích thước của bó cơ này, chúng tôi phát hiện khối lượng cơ của cá thể trưởng thành thấp hơn của cá thể mới rời tổ. Đây có thể là hệ quả của cỡ mẫu nhỏ hoặc là một tác động sinh lý trong quá trình chăm con của thành viên bộ Sẻ. Nghiên cứu của chúng tôi cho thấy các kỹ thuật trực quan hóa với độ phân giải cao rất hữu dụng trong việc phân biệt hình thái ở những nghiên cứu dùng mẫu vật có kích thước nhỏ như chim thuộc bộ Sẻ, nhất là khi có phương pháp lựa chọn mẫu vật kĩ lưỡng.

## Introduction

Throughout ontogeny, shifts in food resource acquisition are common across vertebrates (Duffield and Bull 1998; Whitfield and Donnelly 2006; Wang et al. 2017). Such changes necessarily impact dietary opportunities and behaviors, which can be accommodated by subsequent functional–anatomical changes to the size (e.g., crocodiles; Gignac and Erickson 2015), morphology (e.g., caterpillars to butterflies), and physiology of the feeding system (e.g., birds; Starck 1993), or a combination of each. Birds exhibit contrasting developmental modes, varying from precociality to altriciality, which categorically defines their morphological, behavioral, and physiological characteristics (Starck 1993). A majority of birds exhibit altriciality, including all songbirds (Order Passeriformes) which constitutes 6000 of the ∼10,000 currently recognized species of extant Aves. Altricial birds have young that rely on their parents for food, protection, and thermoregulation (Starck 1993); therefore, as hatchlings and nestlings, songbirds do not manipulate their food, and their feeding apparatus as a whole may not be as fully functional compared with their precocial (e.g., developmentally more advanced) counterparts.

The avian feeding apparatus is part of a kinetic skull held together by ligaments and with motions powered by a series of muscles (Bock, 1964 ; Holliday and Witmer 2007; Bhattacharyya 2013) that enhances food capture (e.g., meat, nectar, and seeds) and aids in manipulation of a wide variety of resources. The highly versatile keratinized rhamphotheca that covers the rostrum and mandible, along with the additional range of motion facilitated by cranial kinesis, is thought to have contributed to modern birds’ global ecological success, exemplified by Passeriformes because its members are exceptionally diverse in their ecologies, diets, and morphologies. Passeriform skull osteology, specifically with respect to the beak (Grant and Grant 1996; Abzhanov et al. 2004; Podos et al. 2004; Clayton et al. 2005; Abzhanov et al. 2006), has been the focus of many studies (Jollie 1958; Warter 1965; Rich et al. 1985; Hernandez et al. 1993; Thomas 2001; James 2004; Abzhanov et al. 2006; Seijas and Trejo 2011; Turker 2012; Donatelli 2013; Previatto and Posso 2015a; Guzzi et al. 2016; Ujhelyi 2016; Lima et al. 2019), but only a small fraction of those studies documented the jaw musculature and other soft tissues that enable kinesis to function (Bock 1985; Nuijens et al. 2000; Genbrugge et al. 2011; Donatelli 2013; Kalyakin 2015; Previatto and Posso 2015b). This is due in part to the diminutive nature of the feeding system, which makes it practically difficult for researchers to work with. The smallest adult passerine bird weighs just 4.2 g, and with the largest member of passerines weighing in at 1.5 kg, not only are the adults small, but the hatchling and fledgling are even smaller. Difficulties presented by small animal size have resulted in a gap in our understanding of how the cranial kinetic system is composed, functioned, and evolved within this highly specialized group of passeriform birds.

Qualitative changes, such as beak length and shape as well as neurocranium shape (Genbrugge et al. 2011) suggest that the size, location, and orientation of jaw musculature associated with cranial kinesis may also shift during ontogeny. In this study, we describe the feeding apparatus of the black-throated finch (Estrildidae: *Poephila cincta*), a seed-eating songbird in the Old-World tropics and Australasia with a short, thick, and conical bill. Juvenile or sub-adult birds rapidly achieve average adult size or larger as a combined result of the steady diet from their parents while not having to perform many energy-consuming tasks, such as flying and active foraging (Starck 1993). Juvenile black-throated finches gain nourishment by begging adults to feed them with their mouths open due to a widely gaping jaw, whereas adults feed by foraging on harder granivorous materials, such as fallen grass seeds and invertebrates and sometimes collecting seeds directly from the seed-heads. Due to these ontogenetic differences in degree and direction of jaw opening, we hypothesize that juveniles and adults may have different muscle configurations that reflect in these life history stage-specific jaw functions. However, we are not able to address this until we identify a practical and effective means to study the delicate and diminutive anatomy of this versatile feeding apparatus. Modern visualization techniques allow for digital quantification of small muscle dimensions without the distortions associated with physical dissection (Sullivan et al. 2019). Therefore, to study these diminutive jaw muscles, we used diffusible iodine-based contrast-enhanced computed tomography (diceCT) and digital dissection (Gignac and Kley 2014; Gignac et al. 2016). We examine the utility of diceCT and digital dissection for small specimens by describing the jaw musculature of two growth stages, adult and fledgling, as well as qualitatively and quantitatively documenting morphology of the jaw adductor chamber and its components. Institutional abbreviations: ISIS: Species360 TZI: Tulsa Zoo, Inc.

## Materials and methods

One fledgling (unknown sex) and one adult female black-throated finch (*P. cincta*) were each acquired as deceased individuals from the Tulsa Zoo, Inc. (Tulsa, OK; Adult *P. cincta* ISIS No. 17981; fledgling *P. cincta* ISIS No. 18032). The fledgling had not molted to adult plumage and was ∼50 days from hatching at time of death based on records kept by the Tulsa Zoo, whereas the adult black-throated finch had mature plumage and was >180 days old at the time of death. The finch specimens were initially stored frozen. All specimens were chemically fixed in 10% neutral buffered formalin for ∼2 weeks. Specimens were then pre-stain CT-scanned to capture skull morphologies, using grayscale thresholding in Avizo_**©**_ version 9.0, 9.3, and 9.5 (Thermo Fisher Scientific, Waltham, MA) to generate skeletal models. Computed tomographic data were collected on a GE phoenix v_**|**_tome_**|**_x s240 high-resolution microfocus CT system (General Electric, Fairfield, CT) at the American Museum of Natural History Microscopy and Imaging Facility (New York, NY) and on a Nikon XTH 225 ST high-resolution microfocus CT system (Tokyo, Japan) at DENTSPLY’s Research and Design Facility (Tulsa, OK). All unstained specimens were scanned at resolutions of <70-μ isometric voxel sizes to obtain the degree of detail necessary to identify bony landmarks. All scanning parameters are listed in **Table 1**.

**Table 1:** Scanning parameters for the adult and fledgling black-throated finch (*P. cincta*) skeletal and contrast-stained data

Specimen	Voxel size	Voltage (kV)	Current (mA)	Exposure time (ms)
Adult skeletal	0.07032915	100	120	200
Adult contrast-stained	0.028752338	180	101	N/A
Fledgling skeletal	0.04857118	90	110	200
Fledgling contrast-stained	0.014593898	147	59	508

After CT scanning for skeletal anatomy, each specimen was soaked in a 3% weight-by-volume (w/v) of Lugol’s iodine (iodine–potassium iodide, I_2_KI) for 10 or 14 days (fledgling and adult, respectively) (Gignac and Kley 2014; Gignac et al. 2016; see **Table 2** for staining information). The solution was refreshed once during the staining period. In an aqueous solution, I_2_KI becomes $I_{3}^{-}$, which binds to fats and sugars in soft tissues (Gignac et al. 2016) and renders those tissues denser than bone. As a result, they are readily visible in X-ray micro-CT images. Once fully stained, specimens were rinsed for 1 h in deionized water to remove excess, unbound iodine, then micro-CT scanned a second time to visualize cranial musculature. In this second scan, the specimens were imaged at resolutions of <29-μ isometric voxel sizes, permitting the detail necessary to distinguish adjacent muscle bellies in the feeding apparatus.

**Table 2:** Specimen staining information; abbreviation: weight-by-volume, w/v

Specimen	Staining solution % w/v	Staining duration (days)
Adult contrast-stained	3	14
Fledgling contrast-stained	3	10

To reconstruct the hard tissue, we reconstructed the pre-stain, skeletal-only image stacks through automatic segmentation, grayscale thresholding, and manual, slice-by-slice touch-up. Head length was measured physically with standard calipers and digitally in Avizo to the nearest millimeter (mm), using the “Measurements” tool. During image-stack processing, we utilized Fiji (National Institutes of Health, Bethesda, MD) to crop, rotate, and re-slice the global axes of the image stack so that they were orthogonal in the standard anatomical planes. Following segmentation of the pre-injection skeletal scans, the diceCT image stacks were processed secondarily. The anatomy of the skull and left-side jaw musculature was manually reconstructed in Avizo based on grayscale value differences. Each muscle was first delineated in the plane that was easiest to discern and evaluate, which in this case was the transverse plane, to differentiate it from adjacent muscle bellies before more thorough segmentation was performed. The initial step generated a tubular schematic of muscles and their attachment points. A more thorough segmentation was then performed on each muscle using a combination of the “Brush” tool and the “Interpolation” tool. When the thorough segmentation in one plane of view was performed, at least one other view was simultaneously monitored in order to identify, corroborate, and confirm the muscle boundaries seen in the primary plane view. Muscle boundaries were determined based on sharp differentiation between grayscale values that usually denotes muscles and dense, unstained connective tissues or muscles and bones/cartilage (see e.g., Gignac and Kley 2014). Segmentation at the muscle boundaries was more conservative, meaning that if voxel grayscale values were determined to be “in-between” those of muscles and bones/cartilage, then those voxels were not included in the segmented muscles. This more thorough segmentation step was then performed on the other planes as well to properly discern additional muscle details such as oblique attachment sites and muscle fibers interdigitation. Due to Lugol’s iodine being a poor contrast-stain for ligaments and other connective tissues, alongside the visibility of muscle fascicles, we are confident that the “denser” grayscale value is indicative of muscle bellies being segmented (Gignac and Kley 2014; Gignac et al. 2016; Supplementary Materials). We measured muscle-volume renderings in Avizo using the “Measurements” tool, and used the archosaur muscle density from Gignac and Erickson (2016) (1.056 g/cm^3^) to calculate the mass of each jaw muscle. The left-side musculature was reconstructed in both specimens for consistency. The following muscles were 3D rendered for the black-throated finch based on work by Bock (1985) and Genbrugge et al. (2011): *Musculus adductor mandibulae externus caudalis* (MAMEC); *M. adductor mandibulae externus rostralis lateralis* (MAMERL); *M. adductor mandibulae externus rostralis medialis* (MAMERM); *M. adductor mandibulae externus rostralis temporalis* (MAMERT); *M. adductor mandibulae externus ventralis* (MAMEV); *M. adductor mandibulae caudalis* (MAMC); *M. depressor mandibulae* (MDM); *M. protractor pterygoideus* (MPP); *M. protractor quadratus* (MPQ); *M. pseudotemporalis profundus* (MPsP); *M. pseudotemporalis superficialis* (MPsS); *M. pterygoideus dorsalis* (MPtD); and *M. pterygoideus ventralis* (MPtV) (see **Table 3**). Even though in accordance with Bock (1985) the pterygoideus muscles are more highly subdivided (i.e., *M. pterygoideus medialis anterior*, *medialis posterior*, and *lateralis*; Bock 1985), we use “dorsalis” and “ventralis” terminology without dividing the muscles into finer partitions to describe the pterygoideus muscles. Image stacks and scan metadata files are available for download through Morphosource.org under project P1079. Anatomical landmarks were labeled with reference to prior descriptions for the Java and medium ground finches by Genbrugge et al. (2011); however, not all anatomical landmarks are present in our rendering because of taxonomic and resolution differences between our samples and reference scans. Following completion of the project, specimens were returned to the Tulsa Zoo by request for incineration per institutional policies.

**Table 3:** Attachment sites of the jaw musculature in the black-throated finch (*P. cincta*) drawn from micro-CT and diceCT data as well as from Bock (1985) and Genbrugge et al. (2011)

Muscles	Attachment sites
*Musculus adductor mandibulae externus profundus* (MAMEP)	Lateroventral surface of the postorbital process; Lateral surface of the lower jaw
MAMEC	Lateral surface in between the postorbital process and the zygomatic process; Dorsomedial and dorsal surface of the lower jaw, near the coronoid process
MAMERL	Expansive surface of the skull between the temporal fossa and otic head of the quadrate; Lateral surface of mandibulae fenestra
MAMERM	Lateral edge of the posterior wall of the orbit; Medial surface of the mandibular fossa and coronoid process
MAMERT	Lateral surface in between the postorbital process and the zygomatic process; superficial to the MAMEC Joins into the MAMERM to reach the lateral surface of the mandibular fossa and coronoid process
MAMEV	Ventral surface of the zygomatic process of the squamosal; Caudodorsomedial and caudodorsal surface of the lower jaw, on the coronoid process
MAMC	The proximal half of the rostrolateral surface of the postorbital process of the quadrate; Caudodorsomedial and caudodorsal surface of the lower jaw, posterior to the coronoid process
MDM	Medioventral surface of the retro-articular process; Lateroposterior surface of the squamosal, parietal, and basisphenoid
MPP	Lateral surface of the interorbital septum and interior surface the allosphenoid; Medial surface of the orbital process of the quadrate and the body of the quadrate
MPQ	Medioposterial surface of the postorbital process and the body of the quadrate
MPsP	Distal rostral surface of the orbital process of the quadrate; Ventromedial surface of coronoid process, medial surface of the mandibular fossa
MPsS	Rostral surface of the quadrate orbital process; Ventromedial surface of coronoid process
MPtD	Anterodorsal surface of the palatine and anterodorsal surface of the pterygoid; Medial surface of the mandibular fossa
MPtV	Posterior surface of the pterygoid; Rostroventraomedial surface of the medial and ventral mandible

## Results

**Fig. 1:**
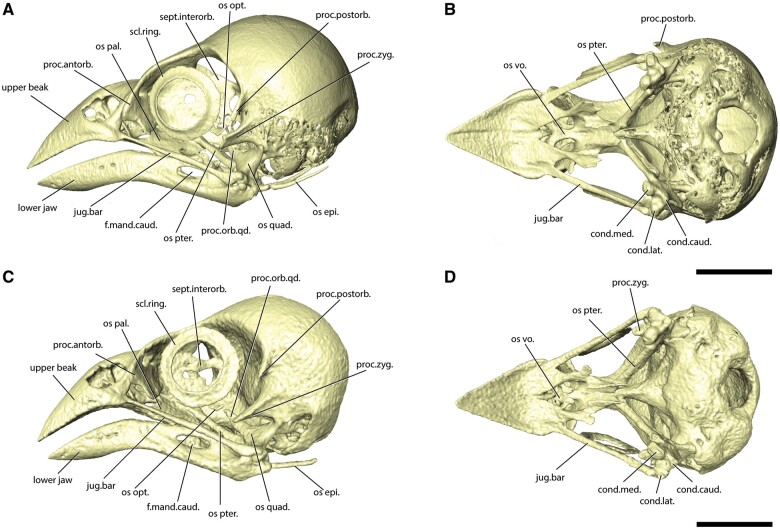
Skull of fledgling black-throat finch in (**A**) lateral view. (**B**) Ventral view without the hyoid and mandible. Skull of adult black-throat finch in (**C**) lateral view.** (D)** Ventral view without the hyoid and mandible. Scale bar represents 5 mm. f.mand.caud., caudal mandibular fenestra; jug.bar, jugal bar; os epi., osseous epihyal; os opt., osseous opticus; os pal., osseous palatine; os pter., osseous pterygoid; os quad., osseous quadrate; proc.antorb., antorbital process; proc.orb.qd., orbital process of the quadrate; proc.postorb., postorbital process; proc.zyg., zygomatic process; scl.ring, sclerotic ring; sept.interorb., interorbital septum.

**Fig. 2:**
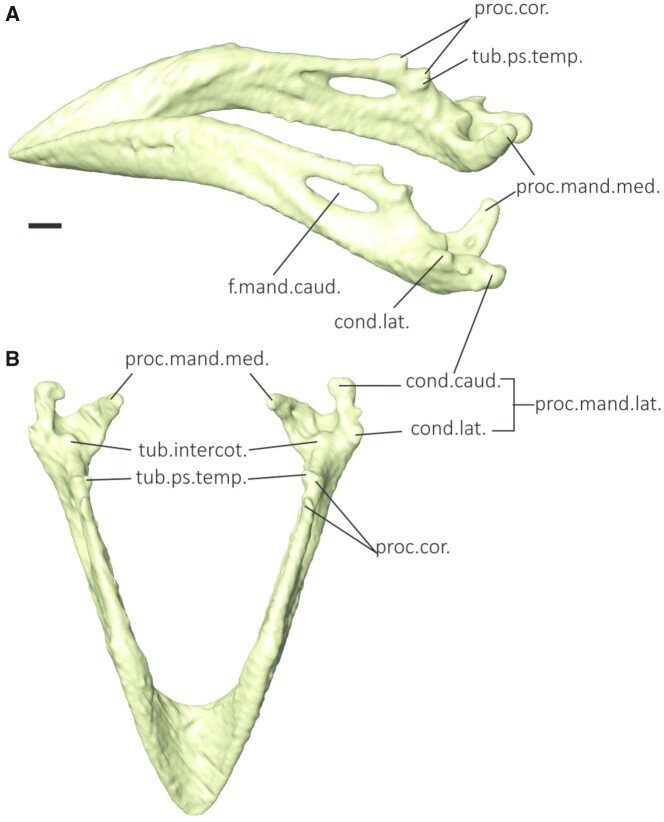
Mandible in the adult black-throat finch. (**A**) Oblique view. (**B**) Dorsal view. Scale bar represents 1 mm. cond.caud., caudal condyle; cond.lat., lateral condyle; proc.cor., coronoid process; proc.mand.lat., lateral mandibular process; proc.mand.med., medial mandibular process; tub.intercot., tuberculum intercotylaris; tub.ps.temp., tuberculum pseudotemporalis.

**Fig. 3:**
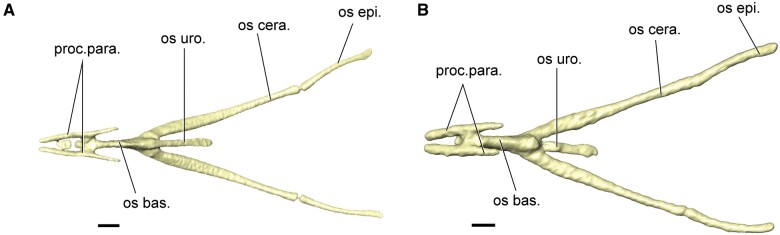
The hyoid in dorsal view. (**A**) Fledgling. (**B**) Adult. Scale bar indicates 1 mm. os bas., osseous basihyal; os cera., osseous cerahyal; os uro., osseous urohyal; proc.para., paraglossal process.

**Fig. 4:**
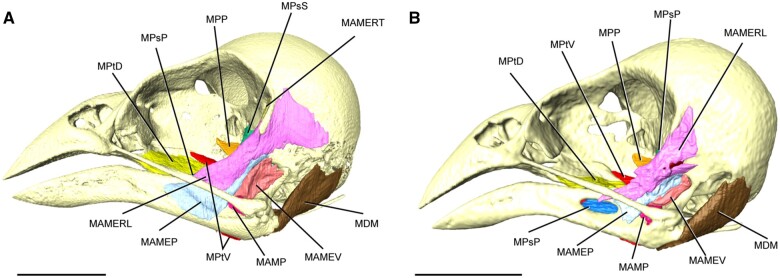
Superficial jaw musculature of black-throat finch in lateral view. (**A**) Fledgling. (**B**) Adult. Scale bar is 5 mm.

The adult skull is 20 mm long from the tip of the beak to the back of the parietal, while the fledgling skull measures 22 mm long. Both the fledgling and adult skulls across the frontal bone from orbit-to-orbit measure 12 mm. The fledgling skull shows less ossification at the posteroventral portion of the skull based on both grayscale and thresholding values in the CT scans (***Figs. 1*** and 4), with many small areas of cartilage and dermal bone likely still composing the posteroventral margin of the cranium. Because cartilage is less mineralized and therefore usually has a lower density value, it is not visualized as well in the CT scans as bone. Both the adult and fledgling skulls show the presence of ossified *os siphonium* (ossified tube connecting the tympanum and the articular air chambers of mandible) and *os opticus* (a partially or completely curved ossified scleral bone surrounding the optic nerve entrance into the eyeball, Fig. 1), which are not present in all birds (Tiemeier 1950). The lower jaw of the adult finch (**Fig. 2**) is morphologically more similar to the Java finch than to the medium ground finch (Genbrugge et al. 2011), especially with the more medial placement of the *tuberculum pseudotemporalis* by the caudal *processus coronoideus*. The hyoid of both the fledgling and adult black-throated finch (**Fig. 3**) are both ossified and the adult hyoid is slightly more robust than the fledgling hyoid.

**Fig. 5:**
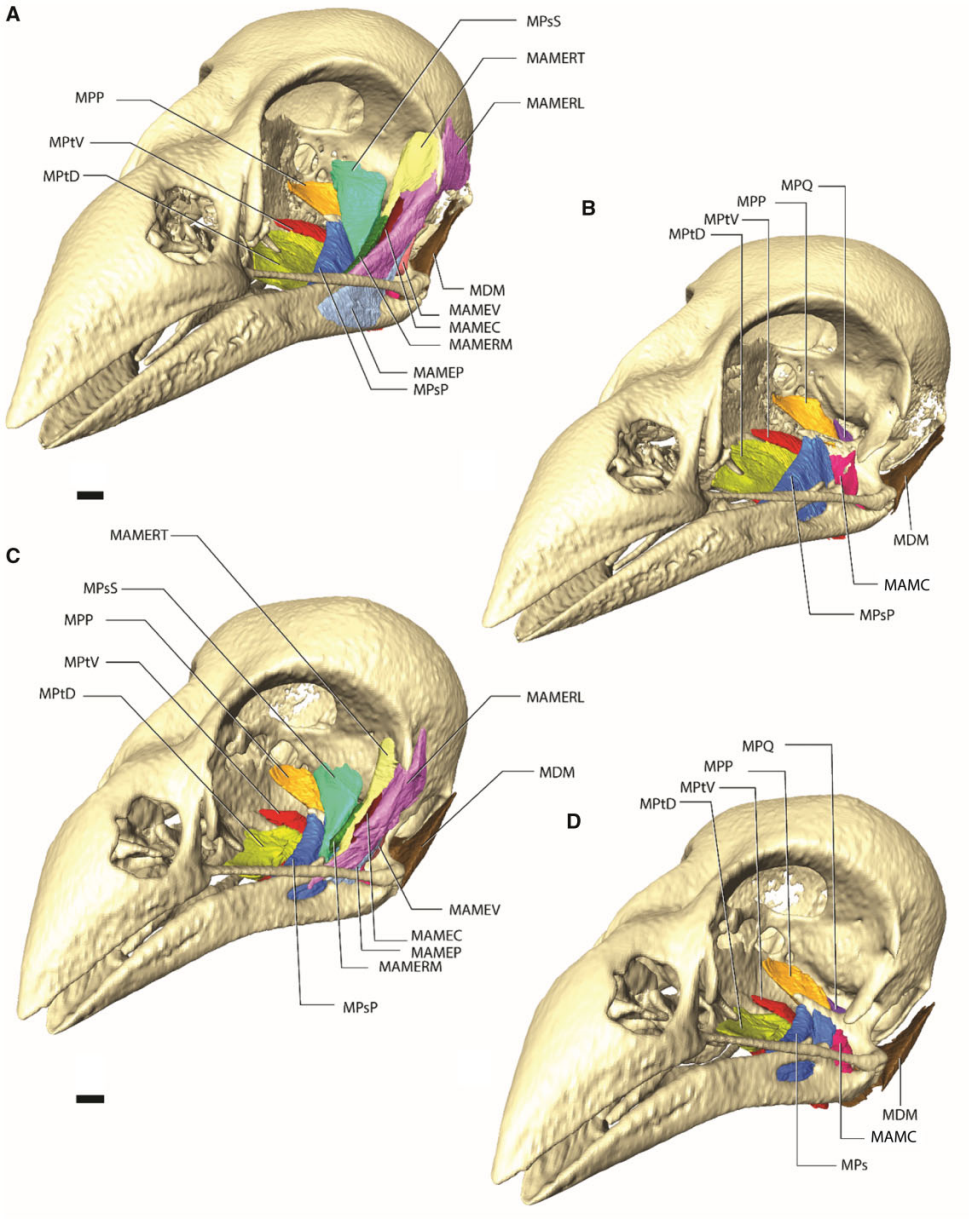
Oblique view of black-throat finch skull. (**A**) All jaw musculature in fledgling. (**B**) Only the profundal jaw musculature in fledgling. (**C**) All jaw musculature in adult. (**D**) Only the profundal jaw musculature in adult. Scale bar represents 1 mm.

All muscle bellies were readily visualized in diceCT datasets. Visualized muscle features included boundaries between muscles, overall muscle morphology, muscle attachment sites, interdigitation of muscles, and (in some muscles) fiber morphologies (see the “Materials and methods” section; ***Figs. 4 and 5***). When compared with the fledgling specimens, the adult had a lower mass of all muscles except for the MDM and the MPP. These differences ranged from −52.8% to −4.98% with MAMERL and MAMERT showing the greatest and least mass deviations, respectively (**Table 4**). The adductor mandibulae externus and pterygoideus muscles make up the two largest muscle groups in mass and volume for both the fledgling and the adult specimen (Table 4). A full breakdown of muscle mass differences is listed in Table 4.

**Table 4:** The jaw muscle measurements of the adult and fledgling black-throated finch (*P. cincta*)

Muscle	Fledgling finch volume (mm^3^)	Fledgling finch mass (mg)	Adult finch volume (mm^3^)	Adult finch mass (mg)	% mass difference
MAMEC	0.97	1.03	0.75	0.79	−23.10
MAMEP	2.31	2.44	1.13	1.19	−51.21
MAMERL	3.46	3.65	1.63	1.72	−52.80
MAMERM	0.86	0.91	0.54	0.57	−37.47
MAMERT	0.92	0.97	0.87	0.92	−4.98
MAMEV	1.22	1.29	1.12	1.19	−37.96
MAMC	2.11	2.23	1.12	1.19	−46.86
MDM	3.65	3.86	4.98	5.26	36.31
MPP	1.26	1.33	1.45	1.53	15.00
MPQ	0.51	0.54	0.46	0.48	−10.20
MPsP	3.07	3.24	2.48	2.62	−19.09
MPsS	2.99	3.16	1.82	1.92	−39.24
MPtD	3.92	4.14	2.49	2.63	−36.59
MPtV	8.61	9.09	7.86	8.30	−8.65

Several of the muscles show multi-pennate morphology, meaning that the sections of muscle fibers within a muscle run in different directions when compared with one or more central tendons. In comparison with the noisy-scrub finch, medium ground finch, and Java finch, the muscles in both the fledgling and adult black-throated finch displayed comparable pennate morphologies except for the MPsS. In the fledgling, the MPsS tri-pennate muscle morphology is seen instead of the bipennate muscle morphology that was reported for this muscle in other species in passerines (Bock 1985; Genbrugge et al. 2011) and in the adult specimen of the current study (Supplementary Materials).

## Discussion

Our diceCT reconstruction of the jaw musculature allowed us to not only discern the minute mass and volume of the soft tissues, but also their morphology and differences between our two growth stages. For example, the tripinnate MPsS in the fledgling may disappear ontogenetically, leading an adult condition of bipinnate muscle morphology commonly seen in more mature individuals. This muscle morphological change has not been documented in other birds and can only be resolved with more densely sampled ontogenetic datasets to determine whether this occurrence was because of individualistic differences or due to actual muscle morphological change. Passeriformes have extremely partitioned and interdigitated muscle groups in comparison to non-passerine birds, and this is found in the black-throated finch as in other finches such as the noisy-scrub finch (Bock 1985), medium ground finch (Genbrugge et al. 2011), and Java finch (Genbrugge et al. 2011). Whether the musculature becomes even more complex and partitioned in other groups or offers some advantages (biomechanical, etc.) over a more simplified muscle arrangement is currently unknown. The increase in musculature complexity can be correlated to an increase in beak dexterity and control. This would effectively allow granivores such as the black-throated finch to easily extract seeds from the seed head or quickly forage for seeds among debris. However, addressing this hypothesis is beyond the scope of the current study and would benefit from an in-depth evaluation across Passeriformes.

Generally, it is expected that older individuals are larger and, therefore, the features of mature individuals should be more massive. However, our adult finch had smaller values for nearly all jaw muscle masses (exception for the MDM and the MPP) in comparison to the fledgling specimen of the same species. Several lines of reasoning supported that the CT image stack of the adult finch did not have any apparent distortion that could be due to muscle shrinkage from the iodine staining. In the scan, the muscle fibers did not appear straightened and “rigid,” and no prominent gaps were apparent between adjacent muscle fiber bundles. The space between the jaw muscles of the fledgling were more prominent than in the adult, but the fledgling’s brain tissues are still flushed against the cranial cavity (see Supplementary Videos S1 and S2) (whereas brain tissues pull away from the cranial cavity as a result of over-staining with salt-rich agents such as Lugol’s iodine; Watanabe et al. 2019, Gignac and Kley 2018). Likewise, not all of the muscle groups showed mass reduction in the adult compared with the fledgling; the MDM showed mass increase. We conclude that if there was muscle shrinkage, it is most reasonable to expect that all muscle groups should show a systematic difference in volume loss ( Vickerton et al., 2013 ; Gignac et al. 2016). As this was not the case, we interpret that muscle-size differences are not due to significant chemical shrinkage (Gignac et al. 2016).

Other factors that could have contributed to this mass difference might be intraspecific differences such that the adult female we sampled was a particularly small individual or that the fledgling was a particularly large individual. Because this species is not reported as sexually dimorphic, we interpret that the sex of the fledgling should not have been a factor regarding overall mass differences. However, breeding conditions might be an important consideration for the adult. Other female songbirds, such as house wrens, have been reported to lose mass during breeding season either for contributing body tissues to offspring production (Freed 1981) or due to decreased foraging time (Norberg 1981; Prince et al. 1981; Newton et al. 1983; Moreno 1989). The adult female specimen sampled for our study may have been breeding when collected, which would have impacted the mass of structures throughout the body. On the other hand, nesting physiology may be an important consideration for the fledgling. For example, finches fledge as they approach adult body size, apparent in our sample as a result of comparable head widths between the fledgling and adult individuals (both 12 mm transverse width across the orbits). Some nesting bird species achieve asymptotic weight while nest-bound because nestlings do not need to expend energy for daily high-cost activities such as flying and foraging (Starck 1993; Stöcker and Weihs 1996). A combination of these factors may explain the less massive jaw musculature that characterizes the adult black-throated finch when compared with the fledgling individual.

Better criteria for specimen collection, including information about sex and in what season the specimen was collected, is stressed for collecting small songbirds because slight differences can contribute to apparently significant changes. Further research should incorporate additional specimens (e.g., at least 10)—including hatchlings, additional fledglings, and male and female adults—to fully explore these findings. These factors are also important considering that digital dissection is a time-consuming process, and the low number of individuals in this study limits some interpretations. Digital dissection speed can be improved by utilizing interpolation tools (Sullivan et al. 2019) along with physical-to-digital comparison via manual dissection. Increasingly improved visualization techniques continue to allow us to better study small specimens, which can lay bare subtle but potentially important differences in exceptionally small, gross anatomical features. Our study demonstrates how these differences can make it difficult to clearly interpret taxon-specific musculoskeletal anatomy. To meaningfully contextualize lilliputian traits, double-digit sampling alongside stricter demographic criteria is requisite to account for possible biological anomalies.

## Supplementary Material

obab007_Supplementary_Data
