# Corrigendum to: Cranial Musculoskeletal Description of Black-Throated Finch (Aves: Passeriformes: Estrildidae) with DiceCT

**DOI:** 10.1093/iob/obac004

**Published:** 2023-01-14

Corrigendum to: Cranial Musculoskeletal Description of Black-Throated Finch (Aves: Passeriformes: Estrildidae) with DiceCT

*Integrative Organismal Biology*, Volume 3, Issue 1, 2021, obab007, https://doi.org/10.1093/iob/obab007

In the originally published version of this manuscript, there was an error in a finch specimen number. In section **Materials and methods**, this should read: “fledgling *P. cincta* ISIS No. 18031).” instead of: “fledgling *P. cincta* ISIS No. 18032).” This error has now been corrected in the article online.

In addition, the display of the Vietnamese abstract has been corrected in the PDF version of the manuscript.

