# Supplementary material for: Cranial Musculoskeletal Description of Black-Throated Finch (Aves: Passeriformes: Estrildidae) with DiceCT
Source: Integr Org Biol. 2021 Apr 30;3(1):obab007. doi: 10.1093/iob/obab007 (PMC8613829; doi:10.1093/iob/obab007)
Supplement: obab007_Supplementary_Data [file obab007_supplementary_data.zip › To et al 2020 Supplemental Figure 1 Caption V2.docx]

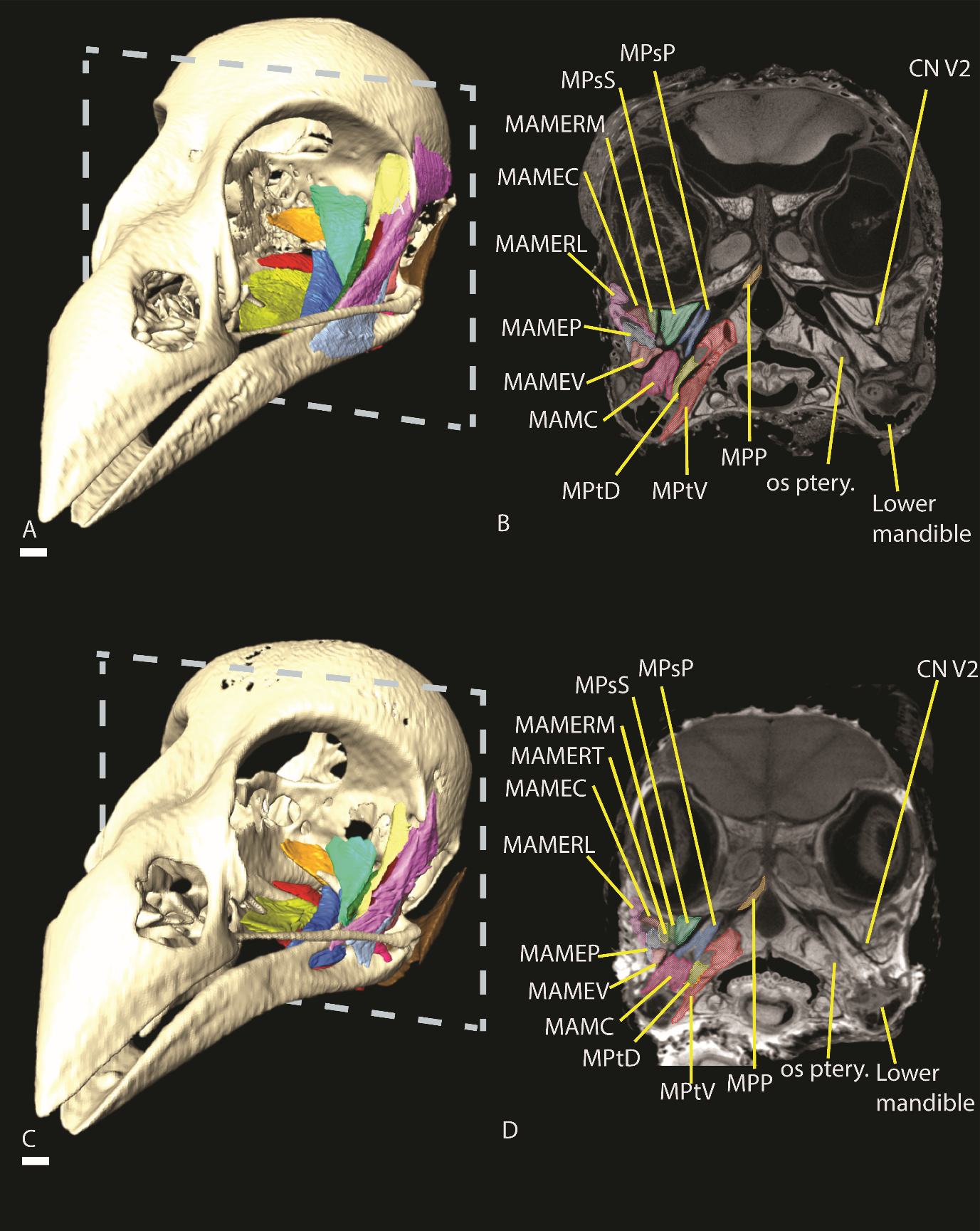
**Supplemental Figure 1:** A. Oblique three-dimensional view of fledgling black-throat finch skull with jaw musculature visualized. B. Transverse cross section of diceCT fledging head with jaw muscles highlighted and key identifying morphological features that aided in the identification jaw muscles indicated. C. Oblique three-dimensional view of adult black-throat finch skull with all jaw musculature. D. Transverse cross section of diceCT adult head with jaw muscles highlighted and key identifying morphological features that aided in the identification jaw muscles indicated. Scale bar represents 1mm; colors of segmented muscles correspond to those in Figures 4 and 5; grayscale value brightness was altered for better segmentation visualization. Abbreviation: CN V2, Cranial nerve V branch 2; MAMEC, *M. adductor mandibulae externus caudalis*; MAMEP, *M. adductor mandibular externus profundus*; MAMERL, *M. adductor mandibular externus rostralis lateralis*; MAMERM, *M. adductor mandibulae externus rostralis medialis*; MAMERT, *M. adductor mandibular externus rostralis temporalis*; MAMEV, *M. adductor mandibular externus ventralis*; MAMC, *M. adductor mandibular caudalis*; MPP, *M. protractor pterygoideus*; MPsP, *M. pseudotemporalis profundus*; MPsS, *M. pseudotemporalis superficialis*; MPtD, *M. pterygoideus dorsalis*; MPtV, *M. pterygoideus ventralis.* [designed for full page width]
