# Supplementary figures and images for: Cranial Musculoskeletal Description of Black-Throated Finch (Aves: Passeriformes: Estrildidae) with DiceCT
Source: Integr Org Biol. 2021 Apr 30;3(1):obab007. doi: 10.1093/iob/obab007 (PMC8613829; doi:10.1093/iob/obab007)

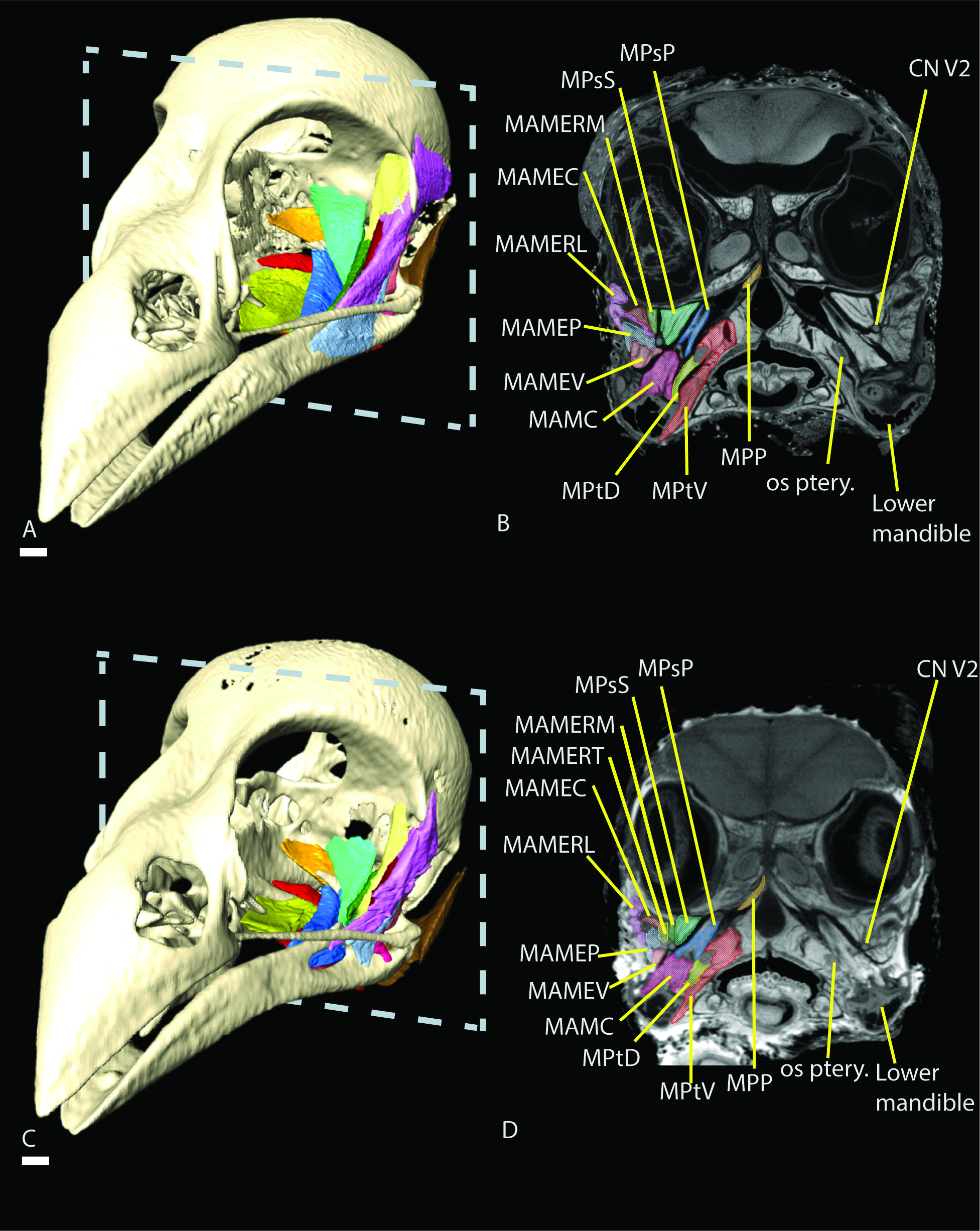

Supplement: obab007_Supplementary_Data [file obab007_supplementary_data.zip › Supplemental Fig_Skull with Muscle Comparison Segmentation.tif]
